# Sequential activation of different pathway networks in ischemia-affected and non-affected myocardium, inducing intrinsic remote conditioning to prevent left ventricular remodeling

**DOI:** 10.1038/srep43958

**Published:** 2017-03-07

**Authors:** Noemi Pavo, Dominika Lukovic, Katrin Zlabinger, Abelina Zimba, David Lorant, Georg Goliasch, Johannes Winkler, Dietmar Pils, Katharina Auer, Hendrik Jan Ankersmit, Zoltán Giricz, Tamas Baranyai, Márta Sárközy, András Jakab, Rita Garamvölgyi, Maximilian Y. Emmert, Simon P. Hoerstrup, Derek J. Hausenloy, Péter Ferdinandy, Gerald Maurer, Mariann Gyöngyösi

**Affiliations:** 1 Department of Cardiology, Medical University of Vienna, Vienna, Austria; 2 Department of Anaesthesiology, Medical University of Vienna, Vienna, Austria; 3 Department of Obstretrics and Gynecology - Molecular Oncology Group, Medical University of Vienna, Vienna, Austria; 4 Department of Surgery, Medical University of Vienna, Vienna, Austria; 5 Department of Pharmacology and Pharmacotherapy, Semmelweis University, Budapest, Hungary; 6 Department of Biochemistry, Faculty of Medicine, University of Szeged, Szeged, Hungary; 7 Department of Biomedical Imaging and Image-guided Therapy, Medical University of Vienna, Vienna, Austria; 8 Institute of Diagnostic Imaging and Radiation Oncology, University of Kaposvar, Kaposvar, Hungary; 9 Swiss Centre for Regenerative Medicine, University of Zurich, Zurich, Switzerland; 10 Division of Surgical Research, University Hospital of Zurich, Zurich, Switzerland; 11 Clinic for Cardiovascular Surgery, University Hospital of Zurich, Zurich, Switzerland; 12 The Hatter Cardiovascular Institute, University College London, London, UK; 13 Cardiovascular and Metabolic Disorders Program, Duke-NUS Graduate Medical School, Singapore, Singapore; 14 Pharmahungary Group, Szeged, Hungary; 15 CeMSIIS - Center for Medical Statistics, Informatics, and Intelligent Systems, Medical University of Vienna, Vienna, Austria

## Abstract

We have analyzed the pathway networks of ischemia-affected and remote myocardial areas after repetitive ischemia/reperfusion (r-I/R) injury without ensuing myocardial infarction (MI) to elaborate a spatial- and chronologic model of cardioprotective gene networks to prevent left ventricular (LV) adverse remodeling. Domestic pigs underwent three cycles of 10/10 min r-I/R by percutaneous intracoronary balloon inflation/deflation in the mid left anterior descending artery, without consecutive MI. Sham interventions (n = 8) served as controls. Hearts were explanted at 5 h (n = 6) and 24 h (n = 6), and transcriptomic profiling of the distal (ischemia-affected) and proximal (non-affected) anterior myocardial regions were analyzed by next generation sequencing (NGS) and post-processing with signaling pathway impact and pathway network analyses. In ischemic region, r-I/R induced early activation of Ca-, adipocytokine and insulin signaling pathways with key regulator STAT3, which was also upregulated in the remote areas together with clusterin (CLU) and TNF-alpha. During the late phase of cardioprotection, antigen immunomodulatory pathways were activated with upregulation of STAT1 and CASP3 and downregulation of neprilysin in both zones, suggesting r-I/R induced intrinsic remote conditioning. The temporo-spatially differently activated pathways revealed a global myocardial response, and neprilysin and the STAT family as key regulators of intrinsic remote conditioning for prevention of adverse remodeling.

Pre-infarction angina, termed “warm-up angina”, increases myocardial resistance to subsequent ischemia, thereby reducing infarct size and mortality^1^. Exposure of the myocardium to single or repetitive brief episodes of ischemia and reperfusion (I/R), before sustained ischemia, induces cardioprotection, which enhances the ability of the myocardium to withstand the next ischemic insult^2^,^3^. Most ischemic preconditioning (IPC) protocols incorporate a sustained coronary occlusion following the IPC-stimulus, causing acute myocardial infarction (AMI). Problematically, the extensive myocardial damage incurred can mask changes to the genomic, proteomic, or metabolic profiles, which would be attributable solely to I/R-linked cardioprotection. Only few reports have employed repetitive I/R (r-I/R) without ensuing AMI to reveal the impact of r-I/R alone, using small animal models with open-chest procedures, or Langendorff perfused hearts *in vitro*^4^. Despite extensive research, translational breakthroughs have not yet been reached^5^, mainly due to the recognition that the experimentally proven powerful IPC-related cardioprotective substances are effective before infarction, and logically should be applied prior to the onset of infarction in the clinical scenario. Therefore, to date, IPC could not achieve practical clinical relevance.

The mechanisms underlying the early (up to 3 hours) and late (from 24 hours to several days post i-I/R) phase of IPC seem diverse: rapidly released and activated transmitter molecules confer beneficial effects in the acute phase, whereas cardioprotection in second windows of protection (SWOP) is instigated by a more complex response that requires de novo synthesis of effector proteins^6^. In contrast to IPC, postconditioning (PostIC) and remote ischemic conditioning (RIPC) are already used in clinical settings, albeit recent meta-analyses revealed a lack of definitive success for both in human procedures. This is partially explained by the application of potentially confounding cardioprotective medications^7^.

Since the term IPC is closely associated with subsequent AMI, we use here the expression r-I/R injury for clear separation of the two distinct phenomena. While IPC (with AMI) represents the warm-up angina followed by ST-segment elevation myocardial infarction (STEMI), r-I/R corresponds to a stable angina in humans.

To date, high-throughput gene analyses have not yet been used for dissecting the effects of IPC or r-I/R in a closed-chest, catheter-based setting in a clinically relevant large animal model, without the masking effect of infarction^7^,^8^, even if this approach is useful for identifying single genes or pathways, or entire pathway networks, and is thus well suited for exploring the complexity of the cardioprotection mechanism^9^.

Here we analyzed the effects of the r-I/R stimulus without subsequent infarction-related injury in a porcine model using next generation sequencing (NGS) combined with pathway network analysis. Our aim was to reveal the complex transcriptomic response that is involved in cardioprotection in ischemic and ischemia-unaffected regions of the heart, and to identify novel genes and networks that are responsible for protection of the heart against I/R. We analyzed the relevant genes and pathways at an early time point after r-I/R injury (early window, trigger of cardioprotection) and 24 h later (second window, performing cardioprotection), and elucidated the time-sequence of the activation of pathways and genes with conditioning function in the ischemic and the ischemia non-affected region, termed “intrinsic remote conditioning” responsible for prevention of LV adverse remodeling.

## Results

### A translational model of r-I/R and IPC

**Figure 1:**
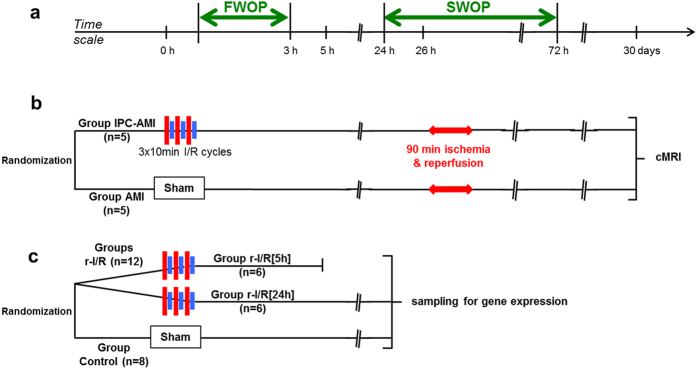
Study design. (**a**) Time scale of the experiments. FWOP: first window of protection; SWOP: second window of protection. (**b**) Efficacy study randomizing domestic pigs into two groups without (Group AMI) or with ischemic preconditioning (IPC; Group IPC-AMI) 26 h before acute myocardial infarction (AMI). Cardiac magnetic resonance imaging (cMRI) at 30 days. (**c**) Gene expression analysis study randomizing pigs into groups with repetitive ischemia/reperfusion (r-I/R) and sacrificing at 5 h (upstream phase of SWOP) and 24 h (SWOP phase) or control.

In contrast to our translational model, small animal open-chest models, with high heart rates differ substantially from the biological scenario presented by the human transient ischemic heart attack. Furthermore, *in vitro* models lack the pathophysiological complexity of the organism. Our assumption, that the typical 30 sec myocardial r-I/R cycles used in rodents for mimicking r-I/R or IPC, would be inadequate in provoking a sustained ischemic response in either pigs or humans, was evidence-based from our previous work showing that indications of adaptive mechanisms can be observed after a minimum of 5 min coronary occlusion in pigs^10^. We found that three 10 min cycles of r-I/R failed to provoke irreversible myocyte injury, as assessed by echocardiography, left ventricular (LV) hemodynamic measurements, necroenzyme release, or microscopic imaging, but induced substantial transcriptomic changes not only within the ischemic injury, but also in the remote myocardial area. Therefore, we induced r-I/R in pigs by using 3 × 10 min I/R by percutaneous balloon inflation/deflation of the mid left anterior descending coronary artery (LAD), followed by recovery of the animals. Sham-operated animals served as control (group control). At the pre-specified (5 h and 24 h) follow-ups (groups r-I/R [5 h] and r-I/R [24 h], respectively), the heart was explanted and the transcriptomics of the ischemia-affected distal anterior, border mid anterior and ischemia-non-affected proximal anterior parts of the heart were determined (Fig. 1). In order to prove the cardioprotective effect of the 3 × 10 min r-I/R stimulus, we have additionally induced AMI 26 h post r-I/R (SWOP phase) (group IPC-AMI) or sham I/R (group AMI) (Fig. 1).

### The r-I/R stimulus induces transient alterations in LV-hemodynamics without persistent myocardial dysfunction

The results of hemodynamic measurements, both invasive and non-invasive, at baseline, directly after the r-I/R stimulus and at follow-up are shown in Supplementary Table S2. As a direct response to the r-I/R stimulus, there was a significant decrease in LV systolic pressures, and an increase in LV end-diastolic pressure and isovolumic relaxation time with normalization at 5 h or 24 h follow-ups.

### Biomarkers and histology

**Figure 2:**
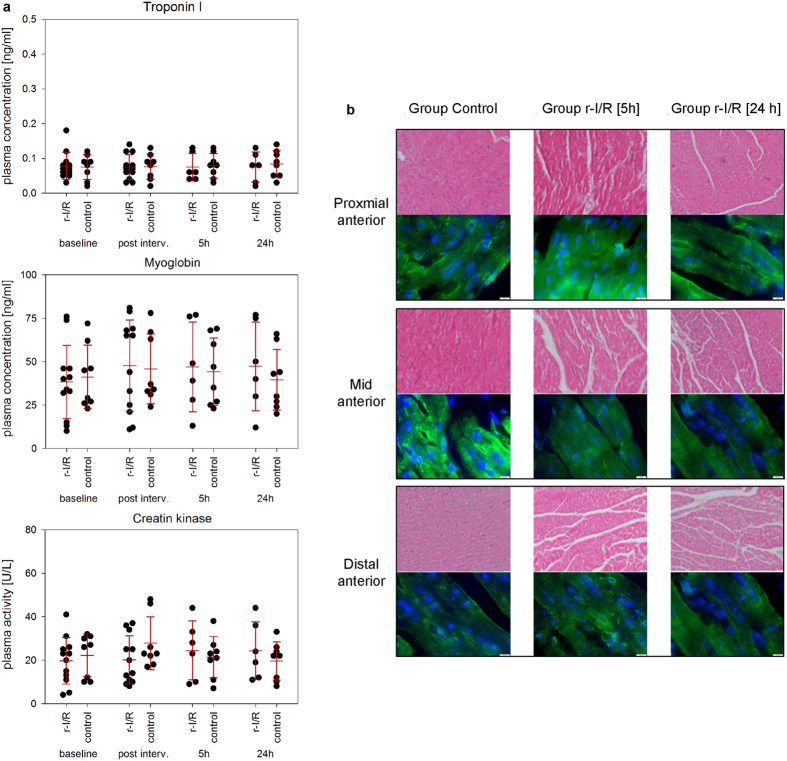
Laboratory data, histology and fluorescence microscopy of the animals with repetitive ischemia/reperfusion (r-I/R) [5 h] and r-I/R [24 h]) or without (group control) r-I/R. (**a**) Serum troponin I (TnI), plasma myoglobin, and serum creatine kinase (CK) at baseline, immediately after r-I/R (post-r-I/R) or sham-r-I/R, at 5 h and 24 h. Animals of groups r-I/R [5 h] and r-I/R [24 h] were pooled as group r-I/R at baseline (n = 12) and post r-I/R (n = 12). Group control: n = 8, 5 h post-r-I/R: n = 6 and 24 h post-r-I/R n = 6. No significant differences between the groups were observed at any time point. Each data point represents the result of one animal, and mean ± s.d. is indicated for each group. (**b**) Haematoxylin-eosin staining (20x magnification) and alpha-actin (green) with DAPI (blue) staining (scale bar 10 μM) of myocardial samples of the proximal, mid and distal anterior regions. No structural changes of the myocardium after r-I/R without subsequent infarction were observed.

The r-I/R stimulus failed to change plasma levels of troponin I, myoglobin or CK (Fig. 2a). Histologic images of the proximal, mid and distal anterior wall regions showed no signs of morphological myocardial injury (Fig. 2b).

### r-I/R leads to time-dependent changes in gene expression compared to non-conditioned hearts

#### Summary of retrieved genes

**Figure 3:**
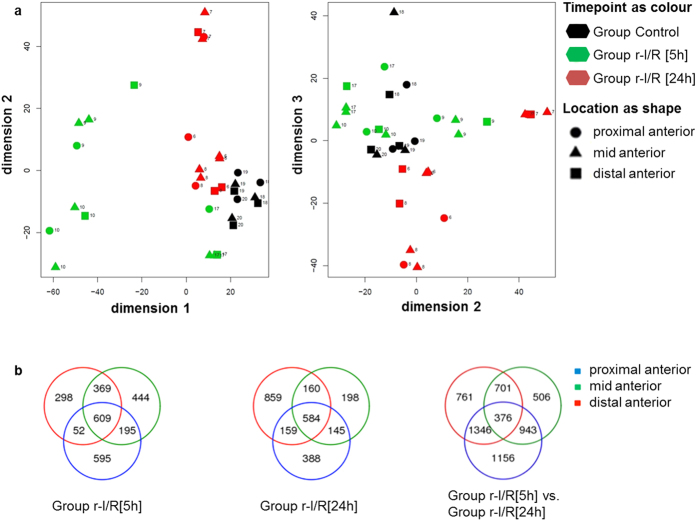
Principal component analysis (PCA) and Venn diagrams of the gene expression analysis. (**a**) Isomaps of 13948 analyzed genes of the myocardial samples from the proximal, mid and distal anterior wall regions from control animals (black) and animals 5 h (r-I/R [5 h], green) or 24 h (r-I/R [24 h], red) after the repetitive ischemia/reperfusion (r-I/R) stimulus. The distal anterior wall was regarded as ischemia/reperfusion-affected area. (**b**) Overlap of the altered gene expression in the proximal, mid and distal anterior regions following the r-I/R stimulus. Venn diagrams show genes with significantly altered expression in the proximal (blue), mid (green) and distal anterior wall (red) regions in the groups r-I/R [5 h] and r-I/R [24 h] compared to controls, and r-I/R [5 h] compared to r-I/R [24 h].

The comparison of the groups r-I/R [5 h] and r-I/R [24 h] with the control group showed that a total of 13758 genes were retrieved by NGS. PCA showed good discriminatory power between the three experimental groups with adequate similarity within the r-I/R groups even though originating from different anterior wall myocardial regions (Fig. 3a). The expressional differences between the r-I/R [5 h] group and controls were greater than between the r-I/R [24 h] group and controls, suggesting more pronounced alterations from normal cell function at 5 h than at 24 h after r-I/R.

Venn diagrams showed significantly altered gene expression between control and r-I/R animals (regardless of the time) and between r-I/R -induced early and late changes for proximal (ischemia-unaffected), mid (border) and distal (ischemia-affected) anterior regions (Fig. 3b). Remarkably, also the proximal region, which was not directly affected by ischemia, showed distinct changes in gene expressions already after 5 h. A total of 5789 genes were differentially expressed between the myocardial samples of the groups r-I/R [5 h] and r-I/R [24 h], equally affecting all three regions.

In the distal anterior wall region, a total of 1328 and 1762 genes were differentially expressed at 5 h and at 24 h, respectively. In the mid anterior region (border zone of ischemia/reperfusion), 1617 and 1087 genes were deregulated 5 h and 24 h post r-I/R, respectively. Regarding the proximal anterior wall region, 1451 and 1276 genes showed differential expression at 5 h and 24 h post r-I/R.

#### Activated pathways and pathway networks of ischemia-affected and non-affected myocardial regions

**Figure 4:**
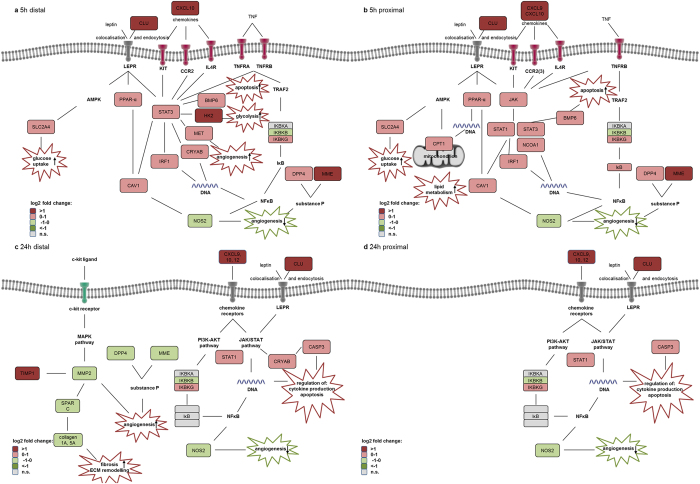
Significantly deregulated gene- and pathway networks at 5 h (group r-I/R [5 h)] and 24 h (group r-I/R [24 h]) after repetitive ischemia/reperfusion (I/R) in the I/R-affected (distal anterior) and –non-affected (proximal anterior) myocardial areas. (**a**) 5 h after r-I/R in the myocardial area subjected to r-I/R. A strong activation of cytokines, their receptors, and downstream signaling molecules of the JAK/STAT and other pathways were found. In particular, strong up regulation of hexokinase 2 (HK2), the hepatocyte growth factor nuclear receptor (MET), interferon regulatory factor 1 (IRF1), alpha-crystallin B chain (CRYAB), and the glucose transporter Glut4 (SLC2A4) resulted, all of which are enhancing either angiogenesis or energy consumption (glucose uptake and glycolysis). Upregulation of peptidases neprilysin (MME) and dipeptidyl peptidase 4 (DPP4), both mediating substance P hydrolysis and inactivation, also indirectly regulates angiogenesis through NO production. (**b**) 5 h after r-I/R in the non-ischemic area. The genes with differential expression in the non-ischemic area are somewhat similar to those found in the ischemic area. A stronger activation of chemokines (CXCL9) and members of the JAK/STAT pathway (JAK3, STAT1) was detected, but no significant regulation of downstream modulators of angiogenesis and glycolysis. This indicates a weaker effect in the remote area, which is not directly affected by ischemia. (**c**) 24 h after r-I/R in the myocardial area subjected to r-I/R. At this time point, a strong up regulation of chemokines was encountered, with a less pronounced differential expression of components of the JAK/STAT pathway (upregulation of STAT1, but not STAT3 or JAK3) or of the NF-κB pathway. Downregulation of critical components of the regulation of collagen production was found, and reduced expression of peptidases MME and DPP4, which enhance angiogenesis through derepression of substance P. (**d**) 24 h after r-I/R in the non-ischemic area. In the non-ischemic area, lower regulatory effects were detected. The alterations in chemokines and the JAK/STAT pathway is similar to the ischemic area at this time point, but no effect on genes that are essential for collagen production or substance P inhibition was present.

Table 1 shows the results of the pathway analyses of the retrieved genes according to the different regions at 5 h and 24 h post r-I/R stimulus compared to controls. Results are presented as SPIA plots with false discovery rates of 5% and 10% for the corresponding pathways (Supplementary Fig. S1). Pathway analysis revealed that there were multiple networks involved at 5 h in the ischemia-affected area, primarily including the calcium signaling, the adipocytokine and insulin signaling pathways. At 24 h (during the late phase of cardioprotection), the immunomodulatory antigen processing and presentation, focal adhesion and extracellular matrix-receptor interaction pathways all were involved in the distal anterior area. The Ca-signaling pathway and the antigen processing and presentation pathways were also activated in the remote myocardial areas at 5 h and 24 h, respectively, indicating decisive roles in intrinsic remote conditioning (Table 1 and Supplementary Fig. S2). Table 2 and Fig. 4 display significantly deregulated genes, which are part of these relevant pathways.

Table 3 and Supplementary Table S3 show a list of relevant selected genes of additional pathways that are significantly involved, showing expressional changes at 5 h and 24 h in the different regions. The genes are listed according to functional classes or established pathways, and changes in expression are indicated according to time points and regions.

#### Major pathways with known role in ischemia/reperfusion

The key regulator STAT3 is overexpressed in the ischemic, but also in the border and non-ischemic areas at 5 h, and mediates the Ca-signaling, adipocytokine and insulin signaling pathways. It is involved in both the PI3K/Akt and the JAK-STAT pathways. Moreover, SHISA2 showed a striking upregulation in both the early and late phases, suggesting the involvement of Wnt-signaling in ischemic preconditioning-induced cardioprotection^11^.

Activation of PI3K and its downstream targets Akt and glycogen synthase kinase 3β (GSK3B), has been demonstrated to be essential for ischemic preconditioning-induced cardioprotection involved in the RISK pathway^12^. Briefly, CREB-1 shows significant upregulation in the early phase, inducing transcriptional regulation of the cAMP pathway. Transcriptional changes in either GSK3A or GSK3B could not be detected in r-I/R-induced injury without subsequent AMI, neither at the early nor at the late phase, suggesting that the involvement of these proteins in cardioprotection does not depend on enhanced transcription. Indeed, most studies imply a post-translational regulation of GSK3B via phosphorylation at serine 9^13^. The PI3K-Akt pathway with the genes found deregulated at 5 h and 24 h post r-I/R is displayed in Supplementary Fig. S3. Our data show that CREB, RTK and ITGA were up- and c-Myb and GF were downregulated. At 24 h, RTK and ITGA showed stable upregulation, while c-Myb displayed baseline levels. We found no modulation of anti-apoptotic Bcl-2, and the transcription and cell survival factor NF-κB showed modest upregulation at 5 h and at 24 h (Supplementary Fig. S3).

The recruitment of STAT3 and the activation of the JAK-STAT pathway play a major role in upregulation of cardioprotective and anti-apoptotic proteins^14^. Other STAT proteins (STAT-1, 5 and 6) have been shown to be upregulated as well, but their precise functional role is not yet fully elucidated^15^. Expression of STAT3 is upregulated in the early phase of r-I/R-induced changes, along with altered expression of several associated proteins, such as TNFα, connective tissue growth factor, osteopontin, tenascin, and several paracrine factors and cytokines^16^. STAT3-KO mice developed cardiac fibrosis, LV remodeling and heart failure^16^. Activation of STAT3 with TNF and its receptors is referred to as the survivor activating factor enhancement (SAFE) pathway^17^. Supplementary Table S4 lists significantly up- or downregulated genes in the RISK and SAFE pathways that have been shown to be upregulated in different phases of IPC (I/R injury with subsequent AMI). Interestingly, r-I/R without subsequent infarction did not result in significant activation of these pathways as a whole, even though some components were deregulated.

#### Regulation of further pathways and genes

In the late phase (24 h), expression of STAT1 was upregulated together with CASP3, both of which have a central role in the activated pathways and are promoting apoptosis. Transcription of CLU (a secreted chaperone that is protective against apoptosis) is significantly elevated both at 5 h and 24 h post r-I/R in ischemic and non-ischemic regions, with an even more pronounced increase at the later time point. CLU has been found to induce ischemic tolerance in neurons^18^, but its role in cardiac r-I/R injury is currently obscure. CLU is secreted in response to cellular stress, promotes cell survival, modulates matrix metalloproteinase expression, and stimulates angiogenesis. Together with leptin, it binds to the leptin receptor, which is subsequently internalized and activates transcriptional pathways including the JAK/STAT pathway^19^. These roles and the marked increase after r-I/R are indicative of an important role in restoring cell function post I/R, and/or indicate a surfeit of CLU to ensure future cardioprotection.

Similarly to a previous experiment by El-Adawi *et al*.^20^, the intrinsic angiotensin II autocrine loop was activated early after r-I/R involving neprilysin (MME), which is recently acknowledged as playing a significant role in LV adverse remodeling. However, without subsequent deep and long-lasting ischemia leading to AMI, the expression of this endopeptidase was almost reduced to baseline levels at 24 h, while even being significantly down regulated in the distal area. MME and the peptidase DPP4 are both upregulated 5 h after r-I/R, but down regulated in the distal zone after 24 h. Among other effects, substance P, which is cleaved by both peptidases^21^, enhances angiogenesis and vasodilatation through release of nitric oxide^22^. The activation of MME and DPP4 at the 5 h time point possibly indicates repression of angiogenesis, followed by de-repression of substance P and angiogenesis in the distal area after 24 h. In addition, the inducible nitric oxide synthase (iNOS2) was reduced at all time points. This indicates a potential cross talk between the MME/DPP4 system and the JAK/STAT pathway (Fig. 4).

Supplementary Table S5 demonstrates a selection of well-documented genes with currently unknown function in the cardiovascular system. The upregulation of expression of some of these genes were stronger than the known cardioprotection-associated genes and further characterization may identify these gene as new potential biomarkers and/or targets.

#### Confirmation of gene expression changes of the anterior wall regions after the r-I/R-stimulus

**Figure 5:**
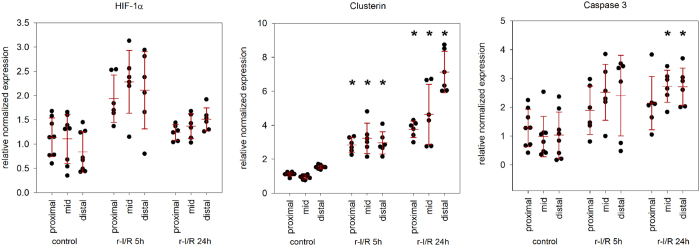
Real-time polymerase chain reaction (RT-PCR) of selected candidate genes in the control (C), and repetitive ischemia/reperfusion groups (r-I/R [5 h] and r-I/R [24 h]) in the different anterior wall regions. A significant increase in clusterin (CLU) was found 5 h after the r-I/R stimulus with a further increase at 24 h. Similar, but less pronounced changes of expression of CASP3 resulted. A trend towards increase in hypoxia-inducible factor-alpha (HIF-1α) resulted. These results verify the NGS data. Each data point represents the result of one animal, and mean ± s.d. is indicated for each group. *p < 0.05 between Group r-I/R [5 h] vs group control in the corresponding myocardial regions.

Quantitative RT-PCR of selected targets confirmed the findings of the NGS analysis (Fig. 5). The r-I/R stimulus resulted in a significant increase of clusterin (CLU) at 5 h with further upregulation at 24 h, and similar, but less pronounced increase in CASP3. Both results are well in line with the sequencing data. We also examined the expression of HIF-1α, the master regulator of cellular oxygen homeostasis. Interestingly, both NGS and qPCR failed to identify a significantly altered transcriptional regulation of HIF-1α in response to the r-I/R stimulus, even though a trend towards increase in HIF-1α expression at 5 h was observed. This could be attributed to short I/R cycles and to the fact that activity of HIF-1α is upregulated primarily by stabilization of the encoded protein, via reduced hydroxylation (mediated by HIF prolyl-hydroxylases), rather than de novo protein synthesis.

### IPC reduces myocardial scar tissue area leading to significantly higher LV EF 30 days after MI during SWOP

In order to prove the cardioprotective effect of the 3 × 10 min r-I/R stimulus, we have additionally induced AMI 26 h post r-I/R (SWOP phase) (group IPC-AMI) or sham I/R (group AMI). The AMI was induced by 90 min catheter-based occlusion of the mid left anterior descending coronary artery followed by reperfusion. The results of cardiac MRI measurements at 30 days are summarized in Table 4. IPC treatment before MI in SWOP resulted in a significant reduction of myocardial scar tissue area, smaller LV ESV and higher LV EF, as well as better segmental wall motion of the ischemia-affected and remote areas, confirming the cardioprotective and reverse remodeling effect of IPC in the late window in this pig model.

## Discussion

### Time-dependent activation of adaptive genomic responses of the myocardium to ischemic stimulus

This is the first study investigating an r-I/R stimulus without subsequent MI in a translational large animal model by the means of NGS, able to retrieve all mRNA transcripts, to identify cardioprotective signaling and the de novo synthesized proteins mediating cardioprotection of the late window. Pathway network and gene expression analyses confirmed time-dependent reprogramming of the ischemic and ischemia non-affected LV gene expression in response to the r-I/R stimulus without MI, with activation of the Ca-signaling, adipocytokine and insulin signaling pathway in the ischemic region early after I/R, and the immunomodulatory pathways at the time of the induced cardioprotection, during the late window of protection. The pathway network analysis provides evidence of spatiotemporal changes in the activation of diverse pathways and complex gene expression profiles of ischemic and non-ischemia-affected zones of the LV. Our data demonstrate that the r-I/R stimulus triggers a distinct adaptive genomic response of the myocardium in a time-dependent manner. Spatiotemporal analysis suggested protective responses in remote, non-ischemia-affected myocardial regions, which might be a target for prevention of ischemic LV remodeling. In contrast to previous experiments, which primarily examined selected genes or groups of related genes, the methods used here, i.e. NGS and pathway network modeling, allowed us to identify the complex responses to I/R in a more comprehensive manner. We analyzed the expressional changes of over 13000 genes by NGS mapping. The results confirmed the reprogramming of the LV in response to the r-I/R injury stimulus. Our data revealed that numerous cardioprotective proteins are necessary for the late windows of protection, which relies on de novo protein synthesis.

### Gene networks and pathways altered by r-I/R

We have identified different networks of pathways at the site of the ischemic injury that were activated sequentially, including Ca-signaling, adipocytokine and insulin signaling pathways at 5 h and several immunomodulatory pathways at 24 h. We found that altered gene expression and related pathway activation were more pronounced at 5 h than at 24 h after r-I/R, underlining the significance of early de-novo protein synthesis, reaching their full effectivity in the SWOP. STAT3 proved to be a key regulator of pathway networks in the early phase, while STAT1 and CASP3 are central nodes in the late phase of cardioprotection. We found an early upregulation of neprilysin both in the ischemic, but also in the non-ischemic region. This endopeptidase has recently been acknowledged as an important signaling molecule of heart insufficiency. Our data suggests also its active role in LV remodeling after I/R injury. In the remote myocardium, Ca-signaling and immunomodulatory pathways were activated at 5 h and at 24 h, indicating intrinsic remote conditioning, in contrast with the neurohumoral mechanism of the classic remote conditioning applying repetitive ischemia in the peripheral muscles. The complexity of the gene regulation patterns revealed in this study suggests that profound biological reactions are triggered by r-I/R and shows that powerful bioinformatics analyses of pathways, gene and protein interactions have the potential to reveal novel targets for therapeutic intervention for clinical relevance.

During conditioning r-I/R cycles, the myocardium activates adaptive mechanisms in an attempt to maintain cell function during actual and delayed hypoxic conditions, and to counterbalance apoptotic signaling by limiting DNA-damage. Unaware of how long the ischemic event will last, the myocardium initially responds in an identical manner to that seen during sustained ischemia. Once a conditioning response is confirmed, the question is to determine the threshold of ischemic burden that will trigger conditioning-induced signaling (instilling ischemic memory) towards irreversible damage.

With reference to previous studies, our NGS data confirms the involvement of pathways and networks already known to be implicated in I/R, such as Ca-signaling, energy metabolism including mitochondrial respiratory chain proteins, myocyte and matrix structure proteins, and stress response proteins including cell fate effectors^12^,^17^,^23^. The strength and novelty of our study lies in its comprehensive approach in identifying *all* interconnected transcripts and interacting pathways, and their regulation with respect to activation, in the ischemic and in the non-ischemic myocardium. The discovery of several activated pathways and upregulated genes in the ischemia-affected and non-affected regions with yet unknown cardiovascular function may serve as a starting point for further research on mitigation of I/R injury and post-ischemic left ventricular remodeling.

### Implications for therapeutic development

Taken together, our data indicates that complex cellular and molecular mechanisms are responsible for cardioprotection through r-I/R, and further regulatory pathways than just the SAFE and RISK pathways are triggered. A number of genes with yet unknown role in I/R have been identified and their precise role and mechanism in cardioprotection may be characterized by further investigations. The profound alterations of several distinct pathways indicate that a multi-targeted therapeutic approach may be feasible. Regarding the concept of systems pharmacology, the precise role of all involved pathways should be taken into account, including the resilience of individual targets to interventional strategies, in order to develop effective therapies. Clearly, I/R is difficult to implement in the clinic. Instead, the elucidation of the functional outcomes may direct the development of novel pharmacologic or gene therapies based on the molecular changes that are responsible for cardioprotection. In this regard, the insight generated by this study facilitates further target characterization and selection to prevent ischemic injury and reverse remodeling of the human heart.

### Study Limitation

Indeed, r-I/R without subsequent infarction did not cause significant myocardial damage and did not elevate the cardiac markers. The 10 min duration of ischemia was selected based on the guidelines on the management of the stable coronary artery disease^24^ defining the stable angina as ischemic pain lasting up to 15 min, and also on our previous observations in pigs^10^ that at least 5 min coronary occlusion is needed to manifest ischemia in intracardiac electrocardiograms. The transition of reversible to non-reversible myocardial ischemia has no clear time-threshold, and depends on a number of different factors, including comorbidities^24^. Our earlier experiments^10^ showed that 10 min r-I/R causes neither permanent low electrical signals nor the segmental wall motion disturbances typical for infarction. However, it results in an incomplete recovery of the myocardial electrical activity even at 24 h post I/R injury, which might also explain the trigger of cardioprotective protein synthesis as a possible mechanism of the second window of cardioprotection in pigs. Although de-novo synthesis of protein is crucial for cardioprotection in SWOP, a complete interpretation of the IPC-induced mechanisms necessitates integration of our NGS findings with proteomic, and other methods that can investigate and verify functional effects and post-translational modifications.

Here, IPC was performed on healthy animals, but atherosclerosis and other cardiovascular co-morbidities may attenuate cardioprotection by IPC^25^. Unfortunately, no animal model exists to simulate the complete palette of human atherosclerotic coronary artery disease. However, as the pig circulation and heart anatomy is very similar to humans, the porcine closed-chest catheter-based coronary intervention model is accepted as the most suitable translational model for human coronary ischemia and treatment.

## Conclusion

We demonstrate for the first time that r-I/R stimuli provokes sequential changes in pathway networks and gene expression profiles not only in ischemic but also in the non-ischemia-affected regions of the myocardium; we introduce the term “intrinsic remote conditioning”, describing an intrinsic protective mechanism against adverse LV remodeling. This experimental approach, using the current, clinically relevant animal model, provides a useful tool for the identification of early and late r-I/R-induced gene expression networks, and may reveal relevant pathways for targeted drug intervention. Concerning the complexity of the response, it is likely that the simultaneous regulation of multiple targets (e.g. mechanisms optimizing cellular metabolism, contractility, inflammation, DNA-repair, and cell survival) is a viable strategy for induction of robust cardioprotection.

## Methods

### Porcine Model of Ischemic Preconditioning

Animal investigations were carried out in accordance with the “Position of the American Heart Association on Research Animal Use,” as adopted by the AHA on November 11, 1984. The study was approved by the Ethics Committee on Animal Experimentation at the University of Kaposvar, Hungary. The study design is displayed in Fig. 1.

Domestic pigs underwent cardiac catheterization (Supplementary Methods). The r-I/R protocol consisted of three repetitive cycles of 10 min I/R via percutaneous balloon occlusion and deflation in the mid left anterior descending coronary artery (LAD) under general anesthesia. Details are described in the Supplementary Methods.

#### (a) Effect of IPC on 30 days cardiac function after acute myocardial infarction (AMI) in SWOP

To prove the effect of IPC in SWOP ten domestic pigs were randomized into two groups with (n = 5, group IPC-AMI) and without (n = 5, group AMI) IPC stimulus (Fig. 1a,b and Supplementary Methods).

#### (b) IPC-induced transcriptomic changes are essential for cardioprotection in SWOP

To assess the time-dependent gene expression alterations as well as pathways and pathway networks that are crucial for triggering cardioprotection and protection in the late window (SWOP), twelve pigs underwent r-I/R, while animals undergoing sham intervention (n = 8) served as controls (group Control). Baseline echocardiography and invasive LV hemodynamic measurements via a pigtail catheter (Cordis, a J&J Company, Fremont, CA) were performed before the preconditioning stimulus. Animals with r-I/R were randomized to groups r-I/R [5 h] (n = 6), or r-I/R [24 h] (n = 6) according to sacrificing time points. At 5 h and 24 h post r-I/R or sham-r-I/R, echocardiography and LV hemodynamics measurements were repeated. Animals were then sacrificed and pig hearts were explanted (Fig. 1c). Myocardial tissue samples from the distal (ischemia-affected), mid (border) and proximal (ischemia non-affected) anterior walls were harvested using biopsy kits (Acu-Punch, Acuderm, Fort Lauderdale, FL).

## Additional Information

**How to cite this article:** Pavo, N. *et al*. Sequential activation of different pathway networks in ischemia-affected and non-affected myocardium, inducing intrinsic remote conditioning to prevent left ventricular remodeling. *Sci. Rep.* **7**, 43958; doi: 10.1038/srep43958 (2017).

**Publisher's note:** Springer Nature remains neutral with regard to jurisdictional claims in published maps and institutional affiliations.

## Supplementary Material

Supplementary Information

## Figures and Tables

**Table 1:** Significantly activated pathways in distal (ischemia-affected), mid (border zone of ischemia) and proximal (non-ischemia affected) anterior wall regions of the heart at 5 h (group r-I/R [5 h]) or 24 h (group r-I/R [24 h]) after repetitive ischemia/reperfusion (r-I/R) without subsequent myocardial infarction.

Location	Name of pathway	ID of pathway	p- value*
***Group r-I*****/*****R [5 h]***			
Distal anterior	Calcium signaling pathway	4020	0.000
	Adipocytokine signaling pathway	4920	0.008
	Insulin signaling pathway	4910	0.009
Mid anterior	Calcium signaling pathway	4020	0.002
	Adipocytokine signaling pathway	4920	0.022
Proximal anterior	Calcium signaling pathway	4020	0.001
***Group r-I*****/*****R [24 h]***			
Distal anterior	Antigen processing and presentation	4612	0.005
	Focal adhesion	4510	0.044
	Extracellular matrix-receptor interaction	4512	0.044
Mid anterior	Antigen processing and presentation	4612	0.000
	Complement and coagulation cascades	4610	0.002
	Graft-versus-host disease	5332	0.004
	Allograft rejection	5330	0.004
	Type I diabetes mellitus	4940	0.004
	Viral myocarditis	5416	0.041
	Cytosolic DNA-sensing pathway	4623	0.042
Proximal anterior	Antigen processing and presentation	4612	0.000
	Complement and coagulation cascades	4610	0.010

^*^Multiplicity correction at false discovery rate 5%.

**Table 2:** Deregulated genes and their expression (fold changes), which are involved in the activated pathway networks in the distal (ischemia-affected), mid (border zone of ischemia) and proximal (not ischemia-affected) anterior wall regions of the heart at 5 h group r-I/R [5 h] or 24 h group r-I/R [24 h] after repetitive ischemia/reperfusion (r-I/R) without subsequent myocardial infarction.

Gene code	Gene name	Group r-I/R [5 h]	Group r-I/R [5 h]	Group r-I/R [5 h]	Group r-I/R [24 h]	Group r-I/R [24 h]	Group r-I/R [24 h]
		Distal	Mid	Proximal	Distal	Mid	Proximal
NCOA1	nuclear receptor coactivator 1	0.13	0.28	**0**.**35**	−0.11	−0.01	0.12
CPT1A	carnitine O-palmitoyltransferase 1	0.57	**0.54**	**0**.**65**	0.04	0.25	0.24
IKBKG	nuclear factor kappa-B essential modulator	**0**.**94**	**0**.**77**	**0**.**99**	**0**.**46**	**0**.**46**	**0**.**48**
PPARA	peroxisome proliferator-activated receptor alpha	**1**.**24**	**1**.**19**	**0**.**89**	−0.33	−0.45	−0.09
SLC2A4	Solute carrier family 2	**0**.**82**	**0**.**55**	**0**.**38**	**−0**.**92**	**−0**.**55**	**−1**.**04**
STAT3	Signal transducer and activator of transcription 3	**0**.**65**	**0**.**64**	**0**.**66**	−0.33	0.03	0.05
TNFRSF1A	tumor necrosis factor receptor superfamily member 1 A	**0**.**50**	**1**.**30**	0.23	0.21	**1**.**33**	0.04
HK2	hexokinase-2	**3**.**36**	1.64	1.09	**−**0.86	**−**1.43	**−**0.49
ALB	albumin	**−1**.**96**	**−1**.**35**	**−1**.**59**	**−**0.22	**−**0.7	**−**0.5
TRAF6	TNF receptor-associated factor 6	0.31	0.41	0.35	**−**0.46	**−**0.03	**−**0.11
CAMKK2	calcium/calmodulin dependent protein kinase kinase 2	**−0**.**58**	**−0**.**51**	**−0**.**45**	**−0**.**8**	**−0**.**66**	**−0**.**77**
MET	hepatocyte growth factor receptor precursor	**0**.**74**	**0**.**58**	0.33	0.26	0.16	0.01
TNFRSF1B	tumor necrosis factor receptor superfamily member 1B	**0**.**48**	0.33	**0**.**35**	0	0.21	0.22
IKBKB	inhibitor of nuclear factor kappa-B kinase subunit beta	**−0**.**83**	**−0**.**8**	**−0**.**89**	**−0**.**66**	**−0**.**33**	**−0**.**51**
NEK6	serine/threonine-protein kinase Nek6	0.21	0.18	0.31	**−**0.47	**−**0.19	**−**0.35
BMP6	bone morphogenetic protein 6	**0**.**53**	0.27	**0**.**57**	0.10	0.40	0.33
MAPK14	mitogen-activated protein kinase 14	0.11	0.22	**0**.**40**	**−**0.23	**−**0.03	0.14
IL4R	interleukin-4 receptor subunit alpha	**0**.**54**	**0**.**50**	**0**.**61**	**0**.**41**	**0**.**41**	0.33
CXCL10	C-X-C motif chemokine 10	**0**.**99**	**1**.**13**	**1**.**23**	**1**.**90**	**2**.**21**	**2**.**37**
HMGB1	high mobility group protein B1	0.14	0.17	**0**.**28**	**−**0.08	**−**0.02	0.15
CLU	clusterin	**2**.**48**	**2**.**3**	**2**.**14**	**3**.**67**	**3**.**71**	**3**.**69**
FAS	tumor necrosis factor receptor superfamily member 6 precursor	**0**.**54**	**0**.**43**	**0**.**66**	0.05	0.17	0.37
CAT	catalase	**−0**.**8**	**−0**.**62**	**−0**.**8**	**−0**.**5**	**−**0.2	**−**0.2
ARNTL	aryl hydrocarbon receptor nuclear translocator-like protein	**1**.**10**	**1**.**29**	**0**.**94**	**−**0.10	**−**0.09	0.16
JAK3	tyrosine-protein kinase	0.35	**0**.**51**	**0**.**72**	0.33	**0**.**64**	0.51
IRF1	interferon regulatory factor 1	**0**.**60**	**0**.**53**	**0**.**76**	**0**.**77**	**0**.**97**	**0**.**88**
CD14	monocyte differentiation antigen CD14	**1**.**11**	**1**.**21**	**1**.**14**	0.41	**0**.**82**	0.53
STAT1	signal transducer and activator of transcription 1	0.29	0.5	**0**.**57**	**0**.**68**	**0**.**83**	**0**.**98**
CAV1	caveolin-1	**0**.**40**	**0**.**38**	**0**.**48**	**−**0.25	**−**0.05	0.17
F2R	proteinase-activated receptor 1 precursor	**0**.**51**	**0**.**40**	**0**.**56**	**−**0.09	0.22	0.32
CXCL9	C-X-C motif chemokine 9	0.50	0.66	**1**.**22**	**1**.**50**	**1**.**93**	**2**.**00**
CCR2	chemokine (C-C motif) receptor 2	**1**.**61**	**2**.**03**	**1**.**89**	0.83	**1**.**40**	1.17
TGFBR2	TGF-beta receptor type-2	**−**0.21	**−**0.02	0.07	**−0**.**73**	**−**0.15	**−**0.27
DPP4	dipeptidyl peptidase 4	**0**.**52**	**0**.**69**	**0**.**82**	**−0**.**47**	0.01	0.22
COL5A1	collagen alpha-1 (V) chain	**0**.**58**	**0**.**62**	0.23	0.38	**0**.**4**	**0**.**4**
SOX9	transcription factor SOX-9	0.29	**−**0.05	0.11	**0**.**91**	**1**.**03**	**−**0.37
SPARC	SPARC precursor	0.02	**−**0.15	0.12	**−0**.**63**	**−**0.25	**−**0.32
TIMP1	metalloproteinase inhibitor 1 precursor	0.32	0.05	0	**1**.**08**	0.55	0.24
CCL2	C-C motif chemokine 2	0.07	0.09	0.35	**0**.**94**	0.77	0.76
CXCL12	stromal cell-derived factor 1	0.01	**−**0.25	0.35	**−0**.**68**	**−**0.22	**−**0.05
NOS2	nitric oxide synthase, inducible	**−0**.**73**	**−0**.**86**	**−0**.**44**	**−0**.**83**	**−0**.**6**	**−0**.**68**
CASP3	caspase-3 subunit	0.42	0.49	0.41	**0**.**64**	**0**.**58**	**0**.**85**
NFKB	NF-kappa-B inhibitor	0.31	**−**0.32	0.8	0.3	0.33	0.34
TYK2	non-receptor tyrosine-protein kinase	**−0**.**74**	**−0**.**57**	**−0**.**48**	**−0**.**48**	**−**0.22	**−**0.28
KIT	mast/stem cell growth factor receptor precursor	**1**	**1**	**0**.**89**	**−0**.**78**	**−**0.6	**−**0.44
MMP2	72 kDa type IV collagenase	0.08	**−**0.11	0.09	**−0**.**65**	**−**0.12	**−**0.25
APOE	apolipoprotein E precursor	0.06	0.31	0.35	**1**.**45**	**−0**.**54**	1.22
GPC3	glypican 3	**−**0.19	**−**0.13	**−**0.44	**−0**.**74**	**−**0.2	**−**0.52
MME	neprilysin	**1**.**15**	**0**.**92**	**1**.**07**	**−0**.**81**	**−**0.09	**−**0.03
ACE	angiotensin-converting enzyme	0.15	0.25	0.08	0.4	**−**0.57	0.01
ND1	NADH-ubiquinone oxidoreductase chain 1	0.05	0.1	**−**0.14	**−**0.5	**−0**.**63**	**−**0.6
SOD1	superoxide dismutase	0.18	0.04	0.05	**−0**.**56**	**−0**.**46**	**0**.**28**
TGFBI	transforming growth factor, beta-induced	**−0**.**29**	**−**0.20	**−**0.17	**−**0.07	0.15	**−**0.09
CRYAB	alpha-crystallin B chain	**0**.**61**	0.38	0.18	**0**.**54**	0.13	0.0
COL1A1	collagen, type I, alpha 2	0.11	**−**0.25	0.23	**−**0.38	**−**0.20	**−**0.3
COL5A2	collagen, type 2, alpha 2	**−**0.08	**−**0.07	0.25	**−0**.**86**	**−**0.18	**−**0.13
TUBA4A	tubulin	**−1**.**47**	**−1**.**44**	**−1**.**73**	**−1**.**39**	**−1**.**61**	**−1**.**87**
SOX9	transcription factor SOX-9	0.29	**−**0.05	0.11	**0**.**91**	**1**.**03**	**1**.**05**
NME1	nucleoside diphosphate kinase A	0.05	**−**0.25	**−**0.03	**1**.**03**	0.14	0.81
PCNA	proliferating cell nuclear antigen	**−**0.02	**−**0.11	0.30	**−0**.**40**	**−**0.28	**−0**.**27**
THBD	thrombomodulin	0.50	**0**.**48**	**0**.**64**	**0**.**67**	**0**.**80**	**0**.**74**
PLAU	urokinase-type plasminogen activator	0.42	**0**.**50**	**0**.**48**	0.45	**0**.**63**	**0**.**56**
ITGAV	integrin alpha-V	0.30	0.37	**0**.**68**	0.58	0.59	**0**.**99**
F3	tissue factor	**−**0.56	0.20	0.82	1.00	**1**.**19**	**1**.**32**
HMOX1	heme oxygenase 1	**−**0.31	**−0**.**67**	**−**0.38	**−**0.62	**−0**.**97**	**−0**.**92**
TNFSF10	tumor necrosis factor ligand superfamily member 10	0.07	**−**0.03	**0**.**58**	0.37	**0**.**77**	**0**.**91**

**Table 3:** Genes with significantly altered expression according to functional groups in distal (ischemia-affected), mid (border zone of ischemia) and proximal (not ischemia-affected) anterior wall regions of the heart at 5 h (group r-I/R [5 h]) or 24 h (group r-I/R [24 h]) after repetitive ischemia/reperfusion (r-I/R) without subsequent myocardial infarction.

Functional group	Gene code	Gene name	Function	Group r-I/R[5 h]	Group r-I/R[5 h]	Group r-I/R[5 h]	Group r-I/R[24 h]	Group r-I/R[24 h]	Group r-I/R[24 h]
				Distal	Mid	Proximal	Distal	Mid	Proximal
Apoptosis/survival	FAIM	Fas apoptotic inhibitory molecule	Protects against death receptor-triggered apoptosis	**−1**.**7**	**−1**.**6**	**−1**.**7**	**−1**.**7**	**−1**	**−1**.**2**
	SHISA2	Shisa family member	Attenuates both FGF and WNT signaling	**2**.**7**	**2**.**5**	**2**.**7**	**1**.**8**	**2**.**4**	**1**.**9**
	TNFRSF12A	Tumor necrosis factor receptor superfamily, member 12 A	Promotes angiogenesis and the proliferation of endothelial cells	**1**.**8**	**1**.**3**	**1**.**8**	**2**	**1**.**2**	**1**.**2**
	CLU/APOJ	Clusterin	Secreted chaperone. suggested role in cell death	**2**.**5**	**2**.**3**	**2**.**5**	**3**.**6**	**3**.**7**	**3**.**7**
	CASP3	Caspase 3, apoptosis-related cysteine peptidase	Apoptosis. necrosis. and inflammation pathway	0.5	0.6	0.5	0.6	0.6	0.9
	ADRA 1B	Adrenoceptor alpha 1B	Regulates growth and proliferation	**2**.**2**	**2**.**1**	**2**.**2**	0.7	0.5	0.3
Oxidative stress	NOS2	Nitric oxide synthase	Generates nitric oxide. high affinity for Ca2+/calmodulin	**−0**.**7**	**−0**.**8**	**−0**.**7**	**−0**.**8**	**−0**.**6**	**−0**.**7**
	GSS	Gluthathione synthethase	Protects cells from oxidative damage by free radicals	**−0**.**8**	**−0**.**6**	**−0**.**8**	0	0.1	0.1
	HSF4	Heat shock transcription factor 4	Activates heat-shock response genes	**−1**	**−0**.**9**	**−1**	**−**0.2	0	**−**0.1
	TRAP1/HSP75	TNF receptor-associated protein 1	Modulates the balance between oxidative phosphorylation and aerobic glycolysis	**−2**.**1**	**−2**.**1**	**−2**.**1**	0	0.1	0
	HIF1-α	Hypoxia inducible factor 1-α subunit	Master transcriptional regulator of the adaptive response to hypoxia	0.1	0.3	0.1	**−**0.5	**−**0.1	0.2
	TXN	Thioredoxin	Thioredoxin reductase. glutaredoxin and glutathione reductase activities. inhibits caspase-3	**−**0.3	0.3	**−**0.4	0.3	**−**0.4	0.2
DNA damage/repair	ERCC4/XPF	Excision repair cross-complementation group 4	Catalytic component of a structure-specific DNA repair endonuclease	**1.7**	**1.7**	**1.7**	0.1	0	0.1
	LIG1	DNA ligase I, ATP-dependent	Integral role in DNA repair and replication	**−0**.**8**	**−0**.**8**	**−0**.**8**	**−0**.**5**	**−**0.2	**−0**.**4**
Ca-signaling	CNN1	Calponin 1, basic, smooth muscle	Implicated in the regulation and modulation of smooth muscle contractions	**2**	**1**.**8**	**2**	**2**	**1**.**3**	0.8
	MYL1	Myosin, light chain 1, alkali, skeletal, fast	ATPase cellular motor protein	**1**.**5**	**1**.**6**	**1**.**5**	0.4	0.5	**−**0.3
	KCNT2	Potassium channel. subfamily T, member 2	Ca^2+^-activated potassium channel	**3**	**3**.**3**	**3**	**1**.**7**	**2**.**6**	**3**
	CAMTA2	Calmodulin binding transcription activator 2	Calmodulin-binding transcription activator	0	0	0	**−0**.**5**	**−0**.**4**	**−0**.**4**
	ATP2B3	ATPase, Ca^2+^ transporting, plasma membrane 3	Critical role in intracellular calcium homeostasis	**0**.**8**	0.5	**0**.**8**	**1**.**3**	0.3	0.5
	CAMKK2	Calcium/calmodulin-dependent protein kinase kinase 2, beta	Phosphorylates the downstream kinases in the calcium/calmodulin-dependent (CaM) kinase cascade	**−0**.**6**	**−0**.**5**	**−0**.**6**	**−0**.**8**	**−0**.**7**	**−0**.**8**
	AGT	Angiotensinogen	Essential component of the renin-angiotensin system (RAS)	**1**.**3**	**1**.**3**	**1**.**3**	0.3	**1.1**	**1**.**2**
Cell structure	MYOC	Myocilin	Role in cytoskeletal function	**−2**.**4**	**−**2.5	**−2**.**4**	**−**0.5	0.4	**−**0.7
	COL4A2	Collagen, type IV, alpha 2	Major structural component of basement membranes	**0**.**6**	**0**.**4**	**0**.**6**	0.3	**0**.**4**	**0**.**4**
	GATA4	GATA binding protein	Myocardial differentation and function	0	0	0	0.2	0.4	0.1
	MEF2c	Myocyte Enhancer Factor 2 C	Role in myogenesis	**−**0.2	**−**0.2	**−**0.2	**−**0.4	**−**0.2	0
	PKC	Protein kinase C	Cell signaling. cell adhesion. cell transformation.	**1**	**1**.**1**	**1**	0.2	0.1	0.7
Immunomodulation	SELL/LECAM 1	Selectin L	Cell surface adhesion protein	**1**.**6**	**1**.**5**	**1**.**6**	0.2	0.7	0.8
	CXCL10	Chemokine (C-X-C Motif) ligand 10	Chemotactic for monocytes and T-lymphocytes	**0**.**9**	**1.1**	**0**.**9**	**1**.**9**	**2**.**2**	**2**.**4**
Protein turnover	CAPN2	Calpain 2, (M/II) large subunit	Calcium-activated neutral protease	**−3**.**4**	**−3**.**9**	**−3**.**4**	**−1**.**6**	**−**1.2	**−**1.6
Energy metabolism	HK2	Hexokinase 2	Couples extramitochondrial glycolysis to intramitochondrial oxidative phosphorylation	**3**	**2**.**6**	**3**	**−**0.9	**−**1.4	**−**0.5
	APOD	Apolipoprotein D	Closely associated with the enzyme lecithin:cholesterol acyltransferase	**2**.**3**	**2**.**3**	**2**.**3**	0.1	0.4	0.5
Cell signaling	MSTN	Myostatin	Negative regulator of skeletal muscle growth	**−1**.**9**	**−**1.1	**−1**.**9**	**−2**.**3**	**−1**	**−1**.**7**
	FGF16	Fibroblast growth factor 16	Required for normal cardiomyocyte proliferation and heart development	**−1**.**9**	**−1**.**8**	**−1**.**9**	0.8	0.8	1.3
	NFKBIB	Nuclear Factor Of Kappa Light Polypeptide Gene Enhancer In B Cells Inhibitor, Beta	Inhibits NF-kappa-B by complexing with and trapping it in the cytoplasm	0.4	**−**0.1	**0**.**8**	0.3	**0**.**8**	0.2
	CTGF	Connective tissue growth factor	Mitoattractant secreted by vascular endothelial cells	**1**.**9**	**1**.**5**	**1**.**9**	0.8	1.1	0.9
	TYK2	Tyrosine kinase 2	Involved in the initiation of type I IFN signaling	**−0**.**7**	**−0**.**6**	**−0**.**7**	**−0**.**5**	**−**0.3	**−**0.3
	CREB	CAMP responsive element binding protein 1	Synchronization of circadian rhythmicity and the differentiation of adipose cells	**0**.**9**	**0**.**6**	**0**.**9**	**0**.**6**	0.4	0.6
	MAPK1/ERK2	Mitogen-activated protein kinase 1	Transduces signals from growth factors and phorbol esters	0.3	0.3	0.3	**−**0.3	**−**0.1	0
	STAT1	Signal transducer and activator of transcription 1	Transcription of IFN-stimulated genes	0.3	0.5	0.3	**0**.**7**	**0**.**8**	**1**

**Table 4:** Cardiac magnetic resonance imaging (cMRI) data collected 30 days after reperfused acute myocardial infarction (AMI) in pigs with and without ischemic preconditioning (IPC), induced by 3 times 10 min ischemia/reperfusion.

cMRI parameter	group IPC-AMI (n = 5)	group AMI (n = 5)	p-value^*^
LVEF, % (IQR)	44.02 (42.31–48.75)	38.60 (37.80–39.80)	**0**.**016**
CO, l/min (IQR)	3.31 (2.96–3.56)	3.00 (2.80–3.00)	0.346
LVEDV, ml (IQR)	62.29 (59.59–73.74)	75.60 (75.10–78.30)	0.117
LVESV, ml (IQR)	39.97 (30.43–42.75)	47.00 (46.10–47.10)	**0**.**047**
LV scar tissue, % (IQR)	5.15 (3.83–8.33)	16.20 (14.10–17.70)	**0**.**028**
LVM, mg (IQR)	69.59 (62.09–76.26)	73.80 (70.40–80.80)	0.251
Infarction transmurality, % (IQR)	55.35 (54.83–61.97)	69.80 (63.50–74.10)	0.175
MO, % (IQR)	0.14 (0.08–0.25)	0.90 (0.50–1.80)	0.053
RVEF, % (IQR)	44.35 (32.53–44.81)	40.70 (39.20–41.50)	0.917
Segmental contraction velocity of ischemia–affected anterior area	14.45 (12.63–16.76)	12.24 (9.98–14.68)	**0**.**044**
Remote anterior area	22.54 (20.39–23.97)	20.12 (18.77–23.55)	0.086

AMI was induced 26 h after IPC (group IPC-AMI) or sham-IPC procedure (group AMI). Data are given in median and interquartile ranges (IQR).

Fonts in bold indicate statistical significance (p < 0.05). *p-values were calculated by the Mann-Whitney-U-test.

LVEF indicates left ventricular ejection fraction; CO, cardiac output; LVEDV, left ventricular end-diastolic volume; LVESV, left ventricular end-systolic volume; LV, left ventricle; LVM, left ventricular mass; MO, microvascular obstruction; RVEF, right ventricular ejection fraction.
