# Supplementary material for: Sequential activation of different pathway networks in ischemia-affected and non-affected myocardium, inducing intrinsic remote conditioning to prevent left ventricular remodeling
Source: Sci Rep. 2017 Mar 7;7:43958. doi: 10.1038/srep43958 (PMC5339807; doi:10.1038/srep43958)
Supplement: Supplementary Information [file srep43958-s1.pdf]

Supplementary Information

Sequential activation of different pathway networks in ischemia-affected and non-affected myocardium, inducing intrinsic remote conditioning to prevent left ventricular remodeling

Noemi Pavo, Dominika Lukovic, Katrin Zlabinger, Abelina Zimba, David Lorant, Georg Goliasch, Johannes Winkler, Dietmar Pils, Katharina Auer, Hendrik Jan Ankersmit, Zoltán Giricz, Tamas Baranyai, Márta Sárközy, András Jakab, Rita Garamvölgyi, Maximilian Y. Emmert, Simon P. Hoerstrup, Derek J Hausenloy, Péter Ferdinandy, Gerald Maurer, and Mariann Gyöngyösi

Table of contents:

Supplementary Methods **2**

In vivo protocols, invasive haemodynamics, echocardiography, cMRI, histology, blood marker analyses, NGS, qPCR, and statistics

References **5**

Supplementary Figures **6**

SPIA plots, Ca-signalling pathway, and PI3Akt pathway

Supplementary Tables **9**

PCR primers, hemodynamic and echocardiographic results, and lists of differentally expressed genes

Supplementary Methods

Porcine Model of Ischemic Preconditioning

The pigs received an intramuscular injection of 12 mg/kg ketamine hydrochloride, 1 mg/kg xylazine and 0.04 mg/kg atropine, with inhalation anesthesia with isoflurane and O_2. After reaching deep anesthesia, pigs were intubated and the anaesthesia was continued with an anesthetic gas mixture of 1.5-2.5 vol% isoflurane, 1.6-1.8 vol% O_2 and 0.5 vol% N_2O. After surgical preparation of the right femoral artery, a 6F introduction sheath (Medtronic Inc, Minneapolis, MN) was placed followed by intra-arterial administration of 200 IU/kg of unfractionated heparin. A 6F coronary catheter (Medtronic Inc, Minneapolis, MN) was placed into the abdominal aorta and selective angiography of the left coronary arteries was performed. After placement of a guidewire (Medtronic Inc, Minneapolis, MN), a coronary balloon dilation catheter (2.75 mm of diameter and 12 mm of length, Medtronic Inc, Minneapolis, MN), was placed into the left anterior descending coronary artery, below the origin of the second diagonal branch. Coronary occlusion was performed using 6 atm inflation pressure, in order to avoid injury of the endothelium of the vessel. Coronary angiography was performed injecting non-ionic contrast media (Takeda, Zürich, Switzerland) to prove the occlusion or the reperfusion of the coronary artery. Electrocardiogram, blood gas and hemodynamics were monitored continuously during the procedure. The study corresponds to the ARRIVE guidelines [S1].

Effect of IPC on 30 days cardiac function after acute myocardial infarction (AMI) in SWOP.

At 26h, during SWOP, the animals underwent myocardial infarction by induction of 90 min sustained ischemia by occlusive inflation of a percutaneous coronary balloon in the mid LAD followed by deflation of the balloon (reperfusion). After 30 days cardiac magnetic resonance imaging (cMRI) was performed to compare the impact of ischemic injury between both groups.

Invasive LV hemodynamics

The following parameters were recorded via a pigtail catheter directly before and after the r-I/R stimulus as well as at follow-up in the r-I/R [5h], r-I/R [24h] and control groups: systolic and diastolic aortic pressure, LV systolic and diastolic pressures (LV SP and LV DP), LV end-diastolic pressures (LV EDP), and a parameter of LV contractility expressed as dP/dt.

Echocardiography

To assess the systolic and diastolic movement of the LV, transthoracic echocardiography was performed at baseline, immediately after the r-I/R stimulus and at 5h and 24h follow-up in the r-I/R [5h], r-I/R [24h] and Control groups. LV diastolic and systolic diameter and fractional shortening were measured, the ejection fraction was determined using Simpson’s method and regional wall motion abnormalities were evaluated visually by blinded investigators. Diastolic parameters such as isovolumetric relaxation time (IVRT), velocity of the E and A waves and their ratio (E/A) were also determined.

cMRI

cMRI was performed at day 30 after reperfused myocardial infarction in the IPC-AMI and AMI groups using a 1.5-T clinical scanner (Avanto, Siemens, Erlangen, Germany) with a phased-array coil and a vector electrocardiogram system (Supplementary Material). The end-diastolic and end-systolic volumes of the LV (LV EDV and LV ESV), LV ejection fraction (LV EF), RV ejection fraction (RV EF), cardiac output (CO), left ventricular mass (LVM), myocardial scar tissue area of the LV and area of transmural myocardial infarction of the LV

were measured. Using the 17-segment model, the LV segmental wall contraction velocities of the ischemia-affected segments (apical-distal anterior and septal anterior) and remote area (proximal anterior) were calculated at the time point of peak ejection (cm/s).

Histology, immunofluorescence analyses, and blood marker analyses

Representative tissue samples from proximal (non-ischemic), mid and distal (ischemia-affected) anterior wall regions were stored in 7.5% neutral buffered formalin, embedded in paraffin, sectioned, and stained with hematoxylin-eosin (HE). Fluorescence immunohistochemistry was performed using alpha actin antibody (Abcam, Cambridge, UK) and counterstaining with DAPI (Abcam). Circulating troponin I (normal value in pigs <0.15 ng/mL), myoglobin (normal range 10-95 ng/mL) and creatine kinase (CK, normal range <100 U/L) were determined at baseline, immediately after the IPC stimulus and at the 5h and 24h follow-up using porcine Cardiac Troponin I Type 3 ELISA (MyBioSource, San Diego, CA, USA), porcine myoglobin ELISA (West Chester, PA) and porcine creatine kinase ELISA (Abcam, Cambridge, UK). Data were compared to that of the sham-operated controls.

Gene Expression Analysis Using Next Generation Sequencing and Real Time PCR (RT-PCR)

Tissue samples from the entire LV were stored in RNAlater (Qiagen, Germany) at -20°C. Total RNA was isolated from the tissue samples using the RNeasy Microarray Tissue Mini Kit (Qiagen, Germany). RNA quality was checked on RNA Nano chips using the Agilent 2100 Bioanalyzer platform (Agilent Technologies).

a) Next generation sequencing (NGS). mRNA of myocardial probes from the basal, mid and apical anterior wall segments of preconditioned and control animals were analyzed. Total RNA (500 ng) were poly-A enriched (NEBNext Poly(A) mRNA Magnetic Isolation Module) and used for library preparation with the NEBNext Ultra Directional RNA Library Prep Kit for Illumina and final libraries were quality controlled on a Fragment Analyzer (Advanced Analytical Technologies) and quantified by digital droplet PCR (QX100™ Droplet Digital™ PCR System, Bio-Rad) and the ddPCR Library Quantification Kit for Illumina (Bio-Rad). Equimolarly pooled samples were sequenced on a HiSeq 2500 to a mean depth of 20.1 million (SD ±5.8 million) paired end reads. After quality check of demultiplexed raw reads by FastQC, reads were mapped to the Sus scrofa genome (Sscrofa10.2) by RNA-Seq Unified Mapper (RUM v2.0.4). Mapped reads were in a range of 91.3% to 93.6% and these mapped reads were counted into the Sus scrofa Ensembl gene model Sscrofa10.2 release 73 (using htseq-count from the HTSeq Python framework to work with high-throughput sequencing data, PMID: 25260700, with “-m union -s reverse”). All further analyses were done in R v3.1.0 (R Core Team, R Foundation for Statistical Computing, ”R: A Language and Environment for Statistical Computing”, 2014, Vienna, Austria, http://www.R-project.org) with R-packages limma v33.2 [S2]., dnet v1.0.6 [S3]., SPIA v2.18.0 [S4]., pathview v1.6.0 [S5]., and biomaRt v2.22.0 [S6]..

Significantly differentially expressed genes were determined with linear models in limma after voom transformation and gene weights calculation between different contrasts with region and individual pigs as confounding factors. Linear models for each gene were fitted and the estimated coefficients and standard errors for all contrasts were computed. Significantly deregulated Kyoto Encyclopedia of Genes and Genomes (KEGG) pathways were analyzed by Signaling Pathway Impact Analysis (SPIA) and illustrated by pathview (node colors represent log_2 fold changes). Biological interpretation of significant gene lists was performed using the Database for Annotation, Visualization and Integrated Discovery (DAVID) v6.7 [S7]. Principal Component Dimensionality reduction of normalized log_2 gene expression values was performed with the non-linear dimensionality reduction method

Isomap [S8], and implemented in the R-package RDRToolbox 1.10.0. Three dimensions were calculated with the modified version of the original Isomap algorithm including the nearest and farthest n-1 neighbors. KEGG pathway enrichment analysis with STRING interaction network was performed to identify the significantly deregulated genes connecting the significantly activated pathways.

b) RT-PCR of Target Genes. mRNA from myocardial probes of the entire LV of representative animals of each group was reverse transcribed to cDNA (Qiagen, Germany) and mRNA expression was quantified by RT-PCR (Applied Biosystems 7500 Real-Time PCR System, Life Technologies, USA). Genes of interest were selected based on suspected involvement in cardioprotection (i.e. HIF-1α, MEF2C, and caspase-3) and based on the results of the NGS analysis (i.e. HK2, ERCC4 and CLU). The primers for the target sequences were designed using Primer3 software (http://primer3.wi.mit.edu/primer3web_help.htm; Microsynth, Switzerland) (Supplemental Table 1). Target gene expression rates were normalized to the geometric means of three housekeeping genes that were selected as endogenous controls due to stable expression (i.e. GAPDH, HPRT1, and PPIA). The relative gene expression level was calculated using the ΔCt method. The expression changes were calculated relative to expression levels in the normal myocardium of control animals that underwent the sham procedure.

Statistics

Continuous data are reported as median and interquartile ranges (IQR). Differences between the groups were tested by the means of two-sided Kruskal-Wallis-Test and the Mann-Whitney-U non-parametric tests, at the alpha level of 0.05. A difference was considered statistically significant at $p<0.05$. Data analyses and interpretations were performed by an experienced observer who was blinded to the randomization and to all study results. Statistical analysis was performed using SPSS 18.0 software (SPSS Inc, USA).

For gene array analysis, to test for all differences between regions of interest (proximal, mid and distal anterior wall regions) and groups (IPC [5h] and IPC [24h] vs. Control), a linear model for each gene was fitted, and the coefficients with standard errors were computed. Moderated t-statistics, F-statistics, and the log-odds of differential expression were computed by empirical Bayes shrinkage of the standard errors towards a common value. A false discovery rate (FDR) below 5% was considered statistically significant. An FDR cut-off of 10% was considered for some contrast, for relevant gene lists for biomedical interpretation. Unsupervised hierarchical cluster analysis was performed using the Euclidean distance as the distance function and the Ward algorithm in R, using centered and scaled log2 expression values. Principal Component Analysis (PCA) was conducted for centered and scaled values.

Supplementary References

S1. Kilkenny C, Browne WJ, Cuthill IC, Emerson M, Altman DG. Improving bioscience research reporting: the ARRIVE guidelines for reporting animal research. PLoS Biol. 2010 Jun 29;8(6):e1000412. doi: 10.1371/journal.pbio.1000412.
S2. Law CW, Chen Y, Shi W, Smyth GK. Voom: Precision weights unlock linear model analysis tools for RNA-seq read counts. Genome biology. 2014;15:R29.
S3. Fang H, Gough J. The 'dnet' approach promotes emerging research on cancer patient survival. Genome Medicine. 2014;6:64.
S4. Tarca AL, Draghici S, Khatri P, et al. A novel signaling pathway impact analysis. Bioinformatics. 2009;25:75-82.
S5. Luo W and Brouwer C. Pathview: an R/Bioconductor package for pathway-based data integration and visualization. Bioinformatics. 2013;29:1830-1981.
S6. Durinck S, Spellman PT, Birney E, Huber W. Mapping identifiers for the integration of genomic datasets with the R/Bioconductor package biomaRt. Nat Protocols. 2009;4:1184-1191.
S7. Jiao X, Sherman BT, Huang da W, et al. DAVID-WS: a stateful web service to facilitate gene/protein list analysis. Bioinformatics. 2012;28:1805-1806.
S8. Tenenbaum JB, de Silva V, Langford JC. A global geometric framework for nonlinear dimensionality reduction. Science. 2000;290:2319-2323.

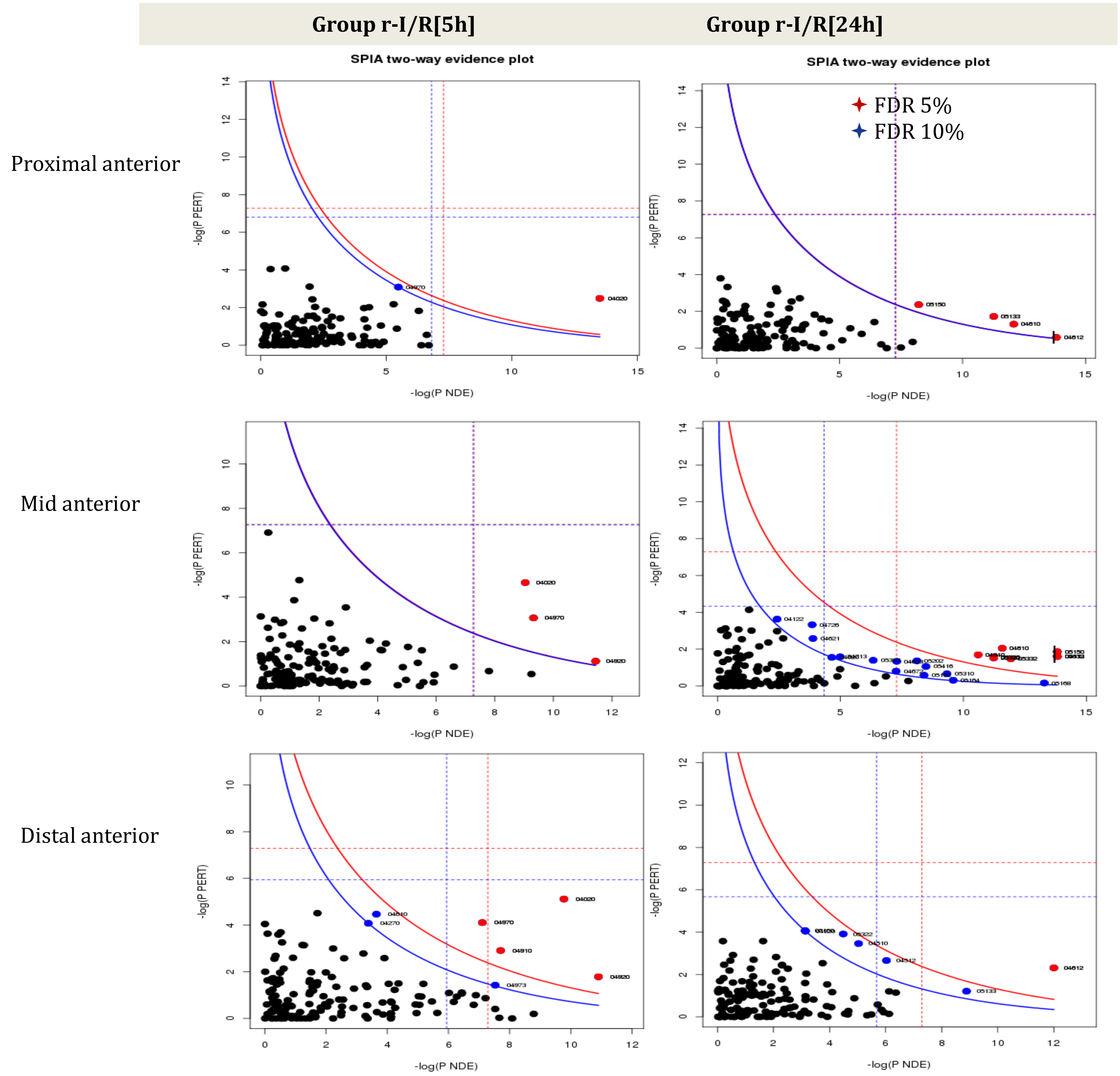

Supplementary Figure S1. SPIA two-way evidence plots for identification of significantly changed cellular pathways.

Pathways with FDR <5 % are highlighted in red, and pathways with FDR between 5 and 10 % in blue.

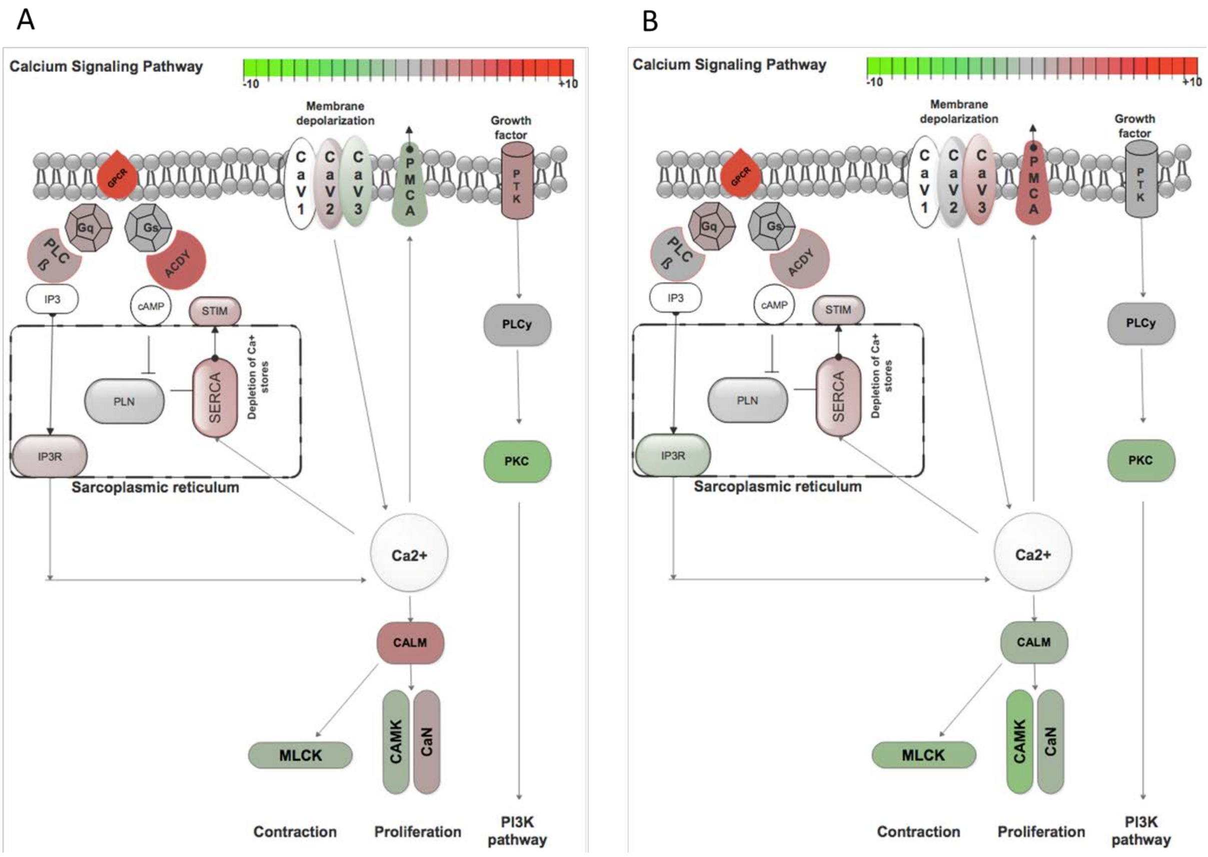

Supplementary Figure S2. Ca-signaling pathway

Genes involved in activated Ca-signaling pathway at 5h (group r-I/R [5h]; A) and 24h (group r-I/R [24h]; B) after repetitive ischemia/reperfusion (r-I/R) in the r-I/R-affected (distal anterior) region. In the ischemic distal anterior region, GPCR, ADCY, SERCA, CALM and IP3R were up- and CAV3, PMCA, PKC, MLCK and CAMK were downregulated at 5h, whereas similar regulation but in a less explicit manner was found at 24h. Relative expression of differentially expressed genes is color-coded (red overexpression, green downregulation).

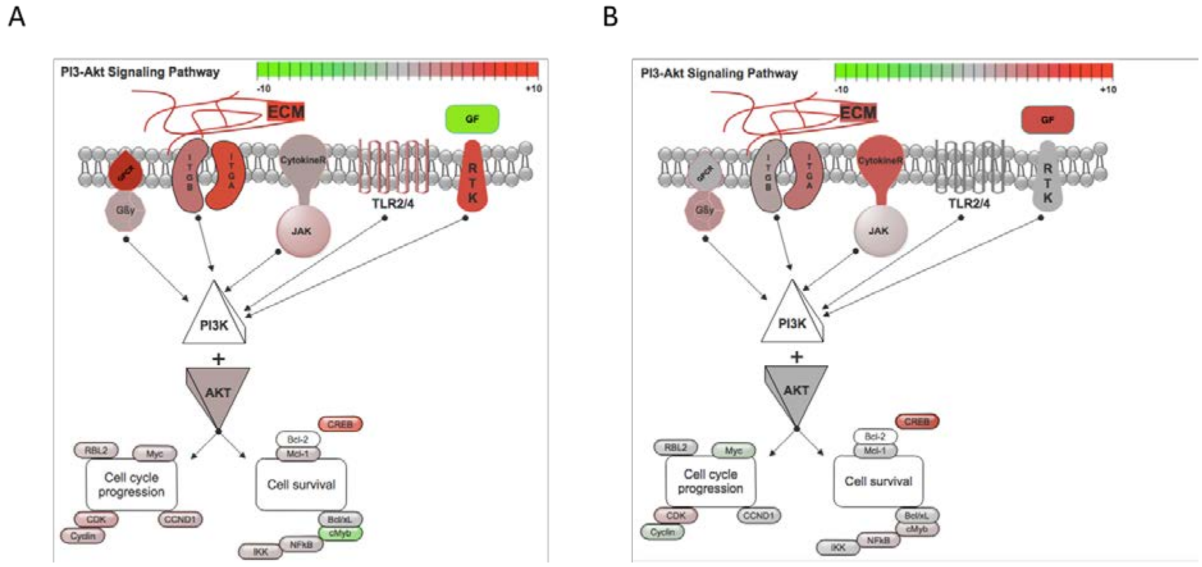

Supplementary Figure S3. PI3-Akt pathway

Genes involved in the activated PI3-Akt-signaling pathway at 5h (group r-I/R [5h]; A) and 24h (group r-I/R [24h]; B) after repetitive ischemia/reperfusion (r-I/R) in the r-I/R-affected (distal anterior) region. Relative expression of differentially expressed genes is color-coded (red overexpression, green downregulation).

Supplementary Table S1. List of primers for RT-PCR.

Gene	Name	Accession no.	forward sequence (5'-3')	reverse sequence (5'-3')	Amplicon [bp]	E [%]
HPRT1	Hypoxanthine phosphoribosyltrans-ferase 1	NM_001032376.2	CCC AGC GTC GTG ATT AGT GA	ATC TCG AGC AAG CCG TTC AG	131	89
PPIA	Peptidylprolyl isomerase A (cyclophilin A)	NM_214353.1	GTC TTC TTC GAC ATC GCC GT	TCC TTT CTC CCC AGT GCT CA	120	95
GAPDH	Glyceraldehyde-3-phosphate dehydrogenase	NM_001206359.1	TCC ACC CAC GGC AAG TTC CAC	ATG TTG GCG GGA TCT CGC TCC T	104	109
HIF1α	Hypoxia inducible factor 1, alpha subunit	NM_001123124.1	ACC TGA GCC TAA CAG TCC CAG TG	TTC TTT GCC TCT GTG TCT TCA GCA A	104	88
CASP3	Caspase 3, apoptosis-related cysteine peptidase	NM_214131.1	GGG ATT GAG ACG GAC AGT GG	TGA ACC AGG ATC CGT CCT TTG	136	95
CLU	Clusterin	NM_213971	CAT GAA GTT CTA CGC GCG TG	AGT AGA AGG GGG AGC TCT GG	92	95

E – primer efficiency.

Supplementary Table S2. Hemodynamic and echocardiographic parameters at baseline, immediately after and at 5h Group r-I/R [5h] and 24h Group r-I/R [24h] following the repetitive ischemia/reperfusion (r-I/R) stimulus and in sham-operated animals.

Variables	baseline (n=12)	sham (n=8)	directly at the end of r-I/R (n=12)	Group r-I/R[5h] (n=6)	Group r-I/R[24h] (n=6)
**Left ventricular haemodynamics**					
max dP/dt [mmHg/s]	868±212	852±62	654±216	587±488	917±207
LV SP [mmHg]	117±13	122±9	90±26*	93±32	109±9
LV DP [mmHg]	0±6	1±1	17±18	3±10	0±2
LV EDP [mmHg]	8±7	7±1	22±19*	11±11	7±11
**Echocardiography**					
LV EF [%]	57±4	63±5	55±12	54±5	54±2
LV EDD [mm]	47±4	44±4	45±3	43±3	45±2
IVRT [msec]	52±6	54±12	72±13*	70±33	52±13

Variables are displayed as mean±sd. p-values were calculated using the Kruskal-Wallis-test; if p-value was <0.05, pairwise comparisons between groups were calculated.
dP/dt indicates time dependent changes of the systolic pressure; LV, left ventricular; SP, systolic pressure; DP, diastolic pressure; EDP, end diastolic pressure; EF, ejection fraction; EDD, end diastolic diameter; IVRT, isovolumetric relaxation time.

*p<0.05 between baseline/sham/24h follow-up post IPC stimulus.

Supplementary Table S3. Additional genes with significantly altered expression according to functional groups in the proximal (not ischemia-affected), mid and distal (ischemia-affected) anterior wall regions of the heart at 5h (Group r-I/R [5h]) or 24h (Group r-I/R [24h]) after repetitive ischemia/reperfusion (r-I/R) without subsequent myocardial infarction.

Functional group	Gene name	Description	Function	Group r-I/R [5h]	Group r-I/R [5h]	Group r-I/R [5h]	Group r-I/R [24h]	Group r-I/R [24h]	Group r-I/R [24h]
				Distal	Mid	Proximal	Distal	Mid	Proximal
Apoptosis/survival	AKTIP	AKT interacting protein	Regulates apoptosis by enhancing phosphorylation and activation of AKT1	-0.6	-0.4	-0.6	0.1	0.0	-0.1
	KIT	V-Kit Hardy-Zuckerman 4 feline sarcoma viral oncogene homolog	Regulates cell survival and proliferation	1.0	1.1	1.0	-0.5	-0.6	-0.8
Oxidative stress	NOSTRIN	Nitric oxide synthase trafficking	Attenuates ENOS-dependent NO production	0.7	0.4	0.7	0.6	0.4	0.7
	NOSIP	Nitric oxide synthase-interacting protein	Negatively regulates nitric oxide production by inducing NOS1 and NOS3	-0.2	-0.3	-0.2	-0.2	0.1	0.1
	CAT	Catalase	Defends against oxidative stress	-0.8	-0.6	-0.8	-0.2	-0.2	-0.5
	TXNRD1	Thioredoxin Reductase 1	May possess thioreductase activity or be involved in apoptosis - depending on isoform	0.6	0.3	0.5	0.3	0.6	0.3
	TXNRD3	Thioredoxin Reductase 3	Thioredoxin reductase, glutaredoxin and glutathione reductase activities	0.6	-0.3	0.9	0.1	0.6	-0.4
	TXNIP	Thioredoxin Interacting Protein	Oxidative stress mediator	-0.2	-0.4	-0.1	-0.4	-0.2	-0.7
DNA damage/ repair	POLH/XPV	Polymerase (DNA directed), Eta	Involved in DNA repair	-0.8	-0.8	-0.8	-0.1	-0.1	-0.3
	PRKDC	Protein kinase, DNA-activated, catalytic polypeptide	Molecular sensor of DNA damage	1.0	1.0	1.0	-0.2	-0.1	-0.1
Ca-signaling	CPNE5	Copine V	Involved in membrane trafficking; exhibits calcium-dependent phospholipid binding properties	0.5	0.7	0.5	2.2	2.0	2.7
	CAB39L	Calcium binding protein 39-Like	Component of a complex that	0.0	0.1	0.0	-0.2	-0.4	-0.4

			binds and activates STK11/LKB1						
	NPPB	Natriuretic peptide precursor B	Cardiovascular homeostasis	1.8	-0.1	1.8	2.6	3.2	6.3
	NPPA	Natriuretic peptide precursor A	Cardovascular homeostasis	1.1	0.9	1.1	1.8	2.7	3.3
	NPR3	Natriuretic peptide receptor 3	Cardiovascular homeostasis	0.7	1.1	0.7	1.2	0.9	0.9
	SCN2B	Sodium channel subunit beta-2	Action potential initiation and propagation in excitable cells	-2.8	-2.4	-2.8	-1.0	-0.6	-1.0
Cell structure	COIL/p80	Coilin	Component of nuclear coiled bodies (CBS)	-0.1	-0.1	-0.1	-0.4	-0.6	-0.8
	MYL1	Myosin, light chain 1, alkali; skeletal, fast	Regulatory light chain of myosin	1.5	1.6	1.5	-0.3	0.5	0.4
	SPTAN1/NEAS	Spectrin, alpha, non-erythrocytic 1	Stabilizes the plasma membrane and organizes intracellular organelles	0.4	0.5	0.4	0.1	0.1	0.0
Immunomodulatory	CCR2	Chemokine (C-C Motif) receptor 2	Receptor for monocyte chemoattractant protein-1, transduces a signal by increasing intracellular calcium ion levels	1.6	1.9	1.6	1.0	1.2	0.7
	FKBP5	FK506 binding protein 5	Immunoregulation	1.8	2.0	1.8	0.4	0.3	-0.1
Protein turnover	UBE2QL1	Ubiquitin-conjugating enzyme E2Q family-like 1	Catalyzes the covalent attachment of ubiquitin to other proteins	1.5	1.7	1.5	2.6	2.8	2.5
	SQSTM1	Sequestosome 1	Binds ubiquitin and regulates the activation of the nuclear factor kappa-B (NF-kB) signaling pathway	0.2	0.1	0.2	0.3	0.4	0.3
Energy metabolism	LIPM	Lipase, family member M	May have an essential function in lipid metabolism	-1.7	-0.9	-1.7	-7.1	-7.4	-7.6
	ENHO	Energy homeostasis associated	Regulates glucose homestasis and lipid metabolism	-2.5	-2.4	-2.5	-1.6	-1.2	-1.3
	ACSM5	Acyl-CoA synthetase medium-chain family member 5	Medium-chain fatty acid:CoA ligase activity with broad substrate specificity	-8.1	-7.2	-8.1	0.5	0.5	0.1
	PRKAG2/AMPK subunit	Protein kinase, AMP-activated, gamma 2 non-catalytic subunit	Important energy-sensing enzyme, regulates de novo biosynthesis of fatty acid and	2.3	2.1	2.3	0.3	0.2	0.2

			cholesterol						
	GYG1	Glycogenin 1	Glycosyltransferase that catalyzes the formation of a short glucose polymer from uridine diphosphate glucose in an autoglucosylation reaction	-0.1	0.1	-0.1	0.3	0.4	0.5
	ALDH2	Aldehyde dehydrogenase 2 family (mitochondrial)	Oxidizes aldehydes to generate carboxylic acids for use in the muscle and heart	-1.1	-1.4	-1.1	0.4	0.4	0.3
Cell signaling	ADAM28	ADAM metallopeptidase Domain 28	May play a role in the adhesive and proteolytic events during lymphocyte emigration	1.5	1.7	1.5	1.3	1.3	1.2
	MPP2	Membrane protein, palmitoylated 2 (MAGUK P55 subfamily member 2)	Associates with the cytoskeleton, suspected to play an important role in signal transduction	-2.2	-2.3	-2.2	-0.3	0.3	-0.2
	MYB	V-Myb avian myeloblastosis viral oncogene homolog	Controls the proliferation and differentiation of hematopoietic progenitor cells	-1.8	-1.1	-1.8	0.1	0.1	0.6
	SIK3	SIK family kinase 3	Serine/threonine kinase activity	0.5	0.5	0.5	0.4	0.4	0.3
	VAV2	Vav 2 guanine nucleotide exchange Factor	Important role in angiogenesis	0.9	0.5	0.9	0.1	0.0	0.0
	RHEB	Ras homolog enriched in brain	Activates mTORC1 signaling	0.5	0.3	0.5	0.3	0.1	0.4
	PIK3R6	Phosphoinositide-3-kinase, regulatory subunit 6	Regulatory subunit of the PI3K gamma complex	-1.0	-1.2	-1.0	-0.1	0.1	0.0
	EFNA1	Ephrin-A1	Important role in angiogenesis	0.2	0.3	0.2	0.3	0.6	0.4
	CD53	CD53 molecule	Efficient formation of myofibers in regenerating muscle	0.6	0.7	0.6	0.4	0.2	-0.1

Supplementary Table S4. Genes with significantly altered expression in specific RISK and SAFE pathways implemented in ischemic preconditioning (IPC) -induced cardioprotection in the distal (ischemia-affected), mid (border zone) and proximal (not ischemia-affected) anterior wall regions of the heart at 5h (Group r-I/R [5h]) or 24h (Group r-I/R [24h]) after repetitive ischemia/reperfusion (r-I/R) without subsequent myocardial infarction.

Functional group	Gene name	Description	Function	Group r-I/R [5h]	Group r-I/R [5h]	Group r-I/R [5h]	Group r-I/R [24h]	Group r-I/R [24h]	Group r-I/R [24h]
				Distal	Mid	Proximal	Distal	Mid	Proximal
RISK pathway	PIK3R6	Phosphoinositide-3-kinase. regulatory subunit 6	Regulatory subunit of the PI3K gamma complex	-1	-1.2	-1	0	0.1	-0.1
	MAPK1/ERK2	Mitogen-activated protein kinase 1	Transduces signals from growth factors and phorbol esters	0.3	0.3	0.3	-0.3	-0.1	0
	PIK3IP1	Phosphoinositide-3-Kinase Interacting Protein 1	Negative regulator of hepatic phosphatidylinositol 3-kinase (PI3K) activity	-0.5	-0.7	-0.5	-0.1	0.2	0.3
	AKTIP	AKT interacting protein	Regulates apoptosis by enhancing phosphorylation and activation of AKT1	-0.6	-0.4	-0.6	-0.1	0	0.1
	CREB	CAMP responsive element binding protein 1	induces transcription of genes in response to hormonal stimulation of the cAMP pathway	0.9	0.6	0.9	0.6	0.4	0.6
	MYB	V-Myb avian myeloblastosis viral oncogene homolog	Controls the proliferation and differentiation of hematopoietic progenitor cells	-1.8	-1.1	-1.8	0.6	0.1	0.1
	PPP2CA	Protein Phosphatase 2. Catalytic Subunit. Alpha Isozyme	negative control of cell growth and division	0.9	0.6	0.6	0.1	0.2	0.3
	GSK3A	Glycogen Synthase Kinase 3 Alpha	negative regulator in the hormonal control of glucose homeostasis. Wnt signaling and regulation of transcription factors	0.1	0	0.1	-0.1	0.1	-0.1
	GSK3B	Glycogen Synthase Kinase 3 Beta	negative regulator in the hormonal control of glucose homeostasis. Wnt signaling and regulation of transcription	0.2	0.5	0.2	0.3	0.3	0.4

			factors						
SAFE pathway	STAT 3	Signal Transducer And Activator Of Transcription 3	part of the JAK-STAT signaling cascade. responses cytokine and growth factor stimuli	0.6	0.7	0.6	-0.4	0	0
	STAT1	Signal transducer and activator of transcription 1	Transcription of IFN-stimulated genes	0.3	0.5	0.3	0.7	0.8	1
	STAT5A	Signal Transducer And Activator Of Transcription 5A	part of the JAK-STAT signaling cascade. responses cytokine and growth factor stimuli	0.6	0.1	0.6	0.8	0.4	0.4
	STAT5B	Signal Transducer And Activator Of Transcription 5B	part of the JAK-STAT signaling cascade. responses cytokine and growth factor stimuli	0.1	0.1	0.1	-0.2	0.2	0
	TNFAIP3	Tumor Necrosis Factor. Alpha-Induced Protein 3	inhibits NF-kappa B activation as well as TNF-mediated apoptosis	0.4	0.5	0.4	0.2	0.6	0.6
	TRAP1/ HSP75	TNF receptor-associated protein 1	Modulates the balance between oxidative phosphorylation and aerobic glycolysis	-2.1	-2.1	-2.1	0	0.1	0
	TNFRSF19	Tumor Necrosis Factor Receptor Superfamily. Member 19	may promote caspase-independent cell death	-1.1	-1.1	-1.1	-1.3	-0.5	-0.9
	TRAF3IP3	TRAF3 Interacting Protein 3	may regulate TRAF3-mediated JNK activation	-0.8	-0.6	-0.8	0	0.1	0.1

Supplementary Table S5. Examples of genes with currently unknown cardiovascular function with significantly altered expression according to functional groups in the proximal (not ischemia-affected), mid and distal (ischemia-affected) anterior wall regions of the heart at 5h (Group r-I/R [5h]) or 24h (Group r-I/R [24h]) after repetitive ischemia/reperfusion (r-I/R) without subsequent myocardial infarction.

Gene name	Description	Function	Group r-I/R [5h]	Group r-I/R [5h]	Group r-I/R [5h]	Group r-I/R [24h]	Group r-I/R [24h]	Group r-I/R [24h]
			Distal	Mid	Proximal	Distal	Mid	Proximal
SKAP2	Src kinase-associated phosphoprotein 2	immune system activation	-2.0	-1.8	-2.0	-2.6	-2.6	-3.0
LYZ	Lysozyme	antibacterial function	1.5	1.9	1.5	2.9	2.9	2.7
CYFIP2	Cytoplasmic FMR1-interacting protein 2	cognitive development	2.0	2.0	2.0	1.5	1.2	1.7
C1QTNF3	Complement C1q tumor necrosis factor-related protein 3	non annotated gene	-2.4	-1.7	-2.4	-0.3	-0.6	-0.9
NELL2	Neural EGFL Like 2	neural cell growth and differentiation, oncogenesis	-1.8	-2.2	-1.8	-0.8	-0.8	-0.9
FGFR4	Fibroblast growth factor receptor 4	influencing mitogenesis and differentiation	-1.8	-1.9	-1.8	0.3	0.4	0.1
CDK15	Cyclin-dependent kinase 1	key player in cell cycle regulation	-1.6	-1.1	-1.6	-0.1	-0.7	-0.6
LEPR	Leptin receptor	regulates adipose-tissue mass	0.4	-0.4	0.4	-1.2	-1.7	-3.7
PTP4A	Protein Tyrosine Phosphatase Type IVA,	cell signaling	1.0	0.8	1.0	0.2	0.1	0.4
ITGA8	Integrin, Alpha 8	cell adhesion, cytoskeletal rearrangement,	1.7	1.6	1.7	0.7	0.7	0.0
INSC	Inscuteable Homolog	cell proliferation, differentiation in nerve system	-2.8	-3.0	-2.8	-1.5	-0.7	-0.8
S100A1	S100 Calcium Binding Protein A1	cell cycle progression	-1.0	-1.3	-1.0	-0.8	-0.6	-0.1
CH242-363D24.1	unknown	unknown	2.2	2.3	2.2	2.0	2.3	2.1
